# Low Grade Neuroendocrine Tumors of the Lung

**DOI:** 10.3389/fonc.2017.00119

**Published:** 2017-06-13

**Authors:** Barbara Melosky

**Affiliations:** ^1^Medical Oncology, British Columbia Cancer Agency – Vancouver Centre, Vancouver, BC, Canada

**Keywords:** neuroendocrine tumors, lung, everolimus, octeotride, Carcinoid, Atypical carcinoid

## Abstract

The lung is the second most common site of neuroendocrine tumors (NETs). Typical and atypical carcinoids are low-grade NETs of the lung. They present a favorable prognosis comported to the more common high-grade NETs. The low- and high-grade NETs require different treatment strategies; effective management of these tumors is essential to prolong survival and to manage the symptoms in patients with secretory or functional tumors. These rare tumors have received little attention and education is needed for treating physicians. This mini-review will concentrate mainly on advanced low-grade lung NETs. The article describes the classification of lung NETs and the diagnostic work-up. Different treatment methods including somatostatin analogs, peptide receptor radioligand therapy, and biologic systemic therapy are discussed. Promising results from recent trials are presented and discussed in the context of the lung primary site.

## Introduction

Neuroendocrine tumors (NETs) are derived from neuroendocrine cells. As these cells exist in many organs embryologically, NETs can initiate in many parts of the body including the gastrointestinal (GI) tract, lung, thymus, and ovary. The lung is the second most common site for NETs after the GI tract, and account for 25% of all NETs (1, 2) and 1–2% of all lung cancers (1, 3, 4).

Neuroendocrine tumors are considered very rare tumors and accurate incidence and prevalence data is difficult to obtain. In 2010, (latest year available) only 315 Canadians were diagnosed with endocrine tumors of all types; the numbers of NETs and even lung NETs are lower still (5). The reported incidence of NETs is increasing, likely due to greater awareness of the disease and better diagnostic capabilities (3). As patients with NETs have a prolonged survival, prevalence rates are high.

Lung NETs are a very heterogeneous group of tumors. They possess varied pathological and clinical features and require different treatment strategies. A spectrum of cell histologies from low grade carcinoid to high-grade small cell malignancies can be observed. Although it is important for the treating physician to understand the disease spectrum of lung NETs, this review will primarily focus on the classification and treatment of low-grade, well-differentiated lung NETs.

Neuroendocrine tumors may secrete biologically active amines or peptides and are often referred to as “functional” or “secretory.” As a result of this secretory activity, patients experience a spectrum of symptoms. Treatment is essential for symptom management and quality of life improvement and may prolong survival. However, as there are only small numbers of patients with lung NETs, evidence for optimal treatment strategies is lacking. The heterogeneous nature of NETs, their rarity and the lack of randomized trials in this disease area, underscores the importance of education in disease management.

## Classifying Lung NETs

The WHO classification of Lung NETs was updated in 2015 and organizes the types of lung NETs on a spectrum, shown in Table 1 (6). A significant change made in the 2015 reclassification was grouping all four NET types into one category. Until this time, large cell and small NETs were separate from the typical carcinoid (TC) and atypical carcinoid (AC) tumors.

**Table 1:** WHO Classification of neuroendocrine tumors (NETs).

NET type	WHO grading (6)	Histology	Mitosis per 2 mm^2^	Presence of necrosis
Low grade (well-differentiated)	G1	Typical carcinoid	<2 (6)	No necrosis
Intermediate grade (well-differentiated)	G2	Atypical Carcinoid	2–10 (6)	Necrosis
High grade (poorly differentiated)	G3	Large cell	>10 (6)	Extensive necrosis
		Small cell		High necrosis

**Figure 1:**
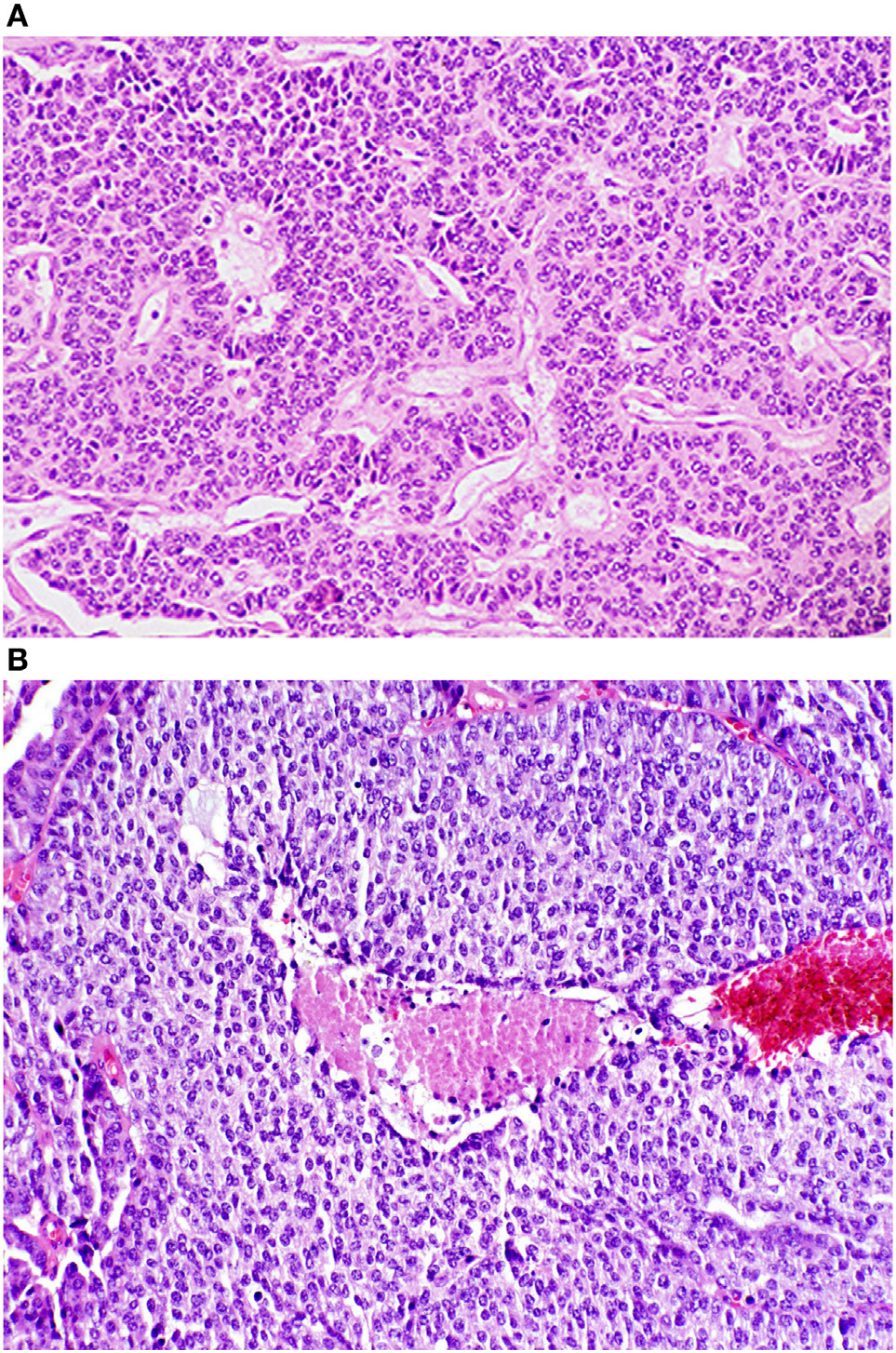
Photomicrographs of typical and atypical pulmonary carcinoid tumors. **(A)** Low power photomicrograph of a typical pulmonary tumor. **(B)** Low power photomicrograph of an atypical pulmonary carcinoid tumor with central necrosis. Reproduced with permission from: Tazelaar HD. Pathology of lung malignancies. In: UpToDate, Post TW (Ed), UpToDate, Waltham, MA. (Accessed on November 2, 2016.) Copyright © 2016 UpToDate, Inc. For more information visit www.uptodate.com.

The WHO classification distinguishes between the low grade (TC and AC) and high grade (large cell neuroendocrine and small cell) tumors. TC tumors are quite bland in their histology, have less than 2 mitoses per 2 mm^2^ and lack any evidence of necrosis. AC tumors can have the same “carcinoid morphology,” but the mitotic rate is increased from 2 to 10 mitoses per 2 mm^2^ and/or may be punctuated with necrotic features. Images of both TC (G1) and AC (G2) NETs are shown in Figures 1A,B, respectively.

Because this review focuses on low-grade NETs, pathology and clinical presentation of high-grade NETs is described only briefly here. As their name implies, large-cell neuroendocrine carcinomas have a large cell size, and a low nuclear to cytoplasmic ratio and frequent nucleoli. The mitotic rate is greater than >10 mitoses per 2 mm^2^ and necrosis is frequently present.

Low-grade NETs include TC and AC tumors. NET G1 or TC tumors, account for 1% of thoracic malignancies with only 10% chance of distant spread (7). NET G2 or AC, account for 0.1% of thoracic malignancies with a 20% chance of distant spread (7). NET G3 large cell NETs have a 4.8% incidence and 50% chance of distant spread, and G3 small-cell NETs have the highest incidence at 13.9%, with the highest chance of distant spread at 70% (7).

The most important point of differentiation for the treating physician is the dichotomous distinction between low grade (carcinoid and AC) and high grade (large cell neuroendocrine and small cell carcinoma) NETs. Prognosis and management differ widely between these two groups. This article will focus on the low- and intermediate-grade NETs. It is important to note that some patients do not fall easily into a discrete category, despite this classification system. Although Ki-67 expression is not validated for use in the lung, it can be used to differentiate the high-grade large cell NETs from the G1/G2 NETs, with crush biopsies or when cells are necrotic (6). Ki-67 is not recommended by the WHO to distinguish the TC from AC tumors (6).

Low-grade lung NETs are subdivided into central or peripheral depending on their site of origin within the bronchial tree. Patients with central lesions may present with symptoms such as hemotypsis (bleeding), wheezing, or airway obstruction. Patients with peripheral disease rarely experience symptoms related to tumor location.

The staging of lung NETs is non-specific and follows the TNM staging of non-NET lung cancers, which follow the current WHO classification (8). This may not be the best staging for this subset of lung malignancies as many lung carcinoid and AC are <3 cm in size (9).

## Procedures for Work up for Advanced Low Grade Lung NET

According to SEER, 12.9% of NET patients present with metastasized tumors at diagnosis (10). Although NETs are slow growing tumors, advanced disease leads to poor survival, and in patients with well-differentiated NETs with distant metastasis, 73% will die within 5 years (1). Liver, bones, and mediastinal lymph nodes are the most common sites of metastasis (11).

Once a diagnosis of advanced low-grade lung NET (carcinoid or AC) is made, a workup to establish disease burden, determine whether the tumor is functional (secretory) or not, and document baseline cardiac status should be initiated. Baseline tests include renal function, calcium and plasma Chromogranin A (12).

For diagnosis, a CT scan of both chest and abdomen should be performed (13). A high resolution CT can be done if contrast is contraindicated. Functional imaging with 111-Indium labeled octreotide is commonly used to establish disease burden and can also indicate whether treatment with peptide receptor radioligand therapy (PPRT) is an option (described in more detail in the Section “Peptide Receptor Radioligand Therapy”) (14, 15). Newer imaging technologies are more accurate, permit tumor staging and better treatment decision making, and can help localize disease. These include ^18^F-dihydroxyphenylalanine (DOPA) positron emission tomography (PET) or preferably, ^68^Ga-DOTATATE PET scan, which also targets somatostatin receptor expression (16, 17).

Functional or secretory NETs may secrete biologically active amines or peptides. Patients may experience a spectrum of symptoms that may include diarrhea, flushing, abdominal pain, hypotension, and vasospasm. Depending on the source, an estimated 10–30% of advanced TC and AC NETs are functional (3, 18).

In patients with functional symptoms, a 24-h urine test for 5-hydroxyindoleacetic acid (5-HIAA) should be performed at baseline (12). High levels of urine 5-HIAA may correlate with the risk of carcinoid heart disease and an attempt to lower it by treating with somatostatin analogs (SSAs) should be made. The 24-h 5-HIAA test should be repeated on disease progression or when a change is therapy is being considered. Because carcinoid complications may occur with time, a baseline echocardiogram should also be performed (19).

## Treatment Modalities for Advanced low Grade NET

For advanced carcinoid and AC patients, treatment is essential for symptom management and quality of life improvement in patients with functional tumors. Treatment may prolong survival in patients with both non-functional and functional tumors. As there are only small numbers of patients with lung NETs, evidence for optimal treatment strategies is lacking. Most NET clinical trials conducted to date have focused on GI NETs, particularly in those of pancreatic (pNET) and midgut origins. Although trial results may be extrapolated, lung NETs deserve individual attention. The heterogeneous nature of NETs, their rarity and the lack of randomized trials in this disease area, underscores the need for trials in this area and the importance of education in disease management.

### Surgery

When TC and AC lung NETs are diagnosed at an early stage, surgical intervention is often curative. TC tumors have excellent 5- and 10-year survival rates of over 90%. This is in contrast to AC tumors where 5-year survival is approximately 70% and 10-year survival is only 50% (20–22).

Regarding adjuvant therapy, the use of chemotherapy with or without radiation has not been well studied and treatment guidelines differ (3, 23, 24). The NCCN guidelines recognize that the role of chemotherapy in the adjuvant setting for typical NET of lung origin is not known (24). However, for stage II or III atypical NET, chemotherapy with or without radiation is recommended (23). The European ENET guideline agrees with this for TC but states that for AC, adjuvant therapy may be considered if nodal disease is found (3).

Surgical treatment may also be considered in patients with advanced or metastatic disease for curative intent or symptom control, depending on the individual patient and site of disease (3).

### Systemic Chemotherapy for Advanced G1 (TC) and G2 (AC) Lung NETs

Patients with low-grade TC and AC lung NETs may respond to chemotherapy, but data are historical and concrete recommendations are not supported. Multiple cytotoxic drug combinations have shown degrees of activity in lung NETS, although there is a lack of consensus regarding standard therapy.

### SSAs for Advanced Low Grade NET

Patient with functional tumors need appropriate treatment to control the functional symptoms of diarrhea, flushing, abdominal pain, hypotension, and vasospasm. In addition to symptom control, randomized trials have demonstrated the benefits in slowing disease progression (25, 26). These will be described in more detail below.

Somatostatin receptors are often overexpressed on the surface of low-grade lung NETs (27). SSAs bind to the somatostatin receptor, blocking the release of peptides and amines, and thus help to control symptoms. The two SSAs currently available in clinical practice for advanced low-grade NETs are octreotide and lanreotide. Pasareoide is a third SAA, not yet in clinical use but currently being tested in a lung NETs clinical trial (28).

Octreotide is available as both intermediate acting subcutaneous (SC) and long-acting release (LAR) formulations. A 30-mg IM dose of octeotride-LAR can be repeated every 4 weeks, and increased by 10 mg increments up to an octeotride-LAR dose of 60 mg. At this dose, most receptors are saturated and increasing it beyond has little benefit (29). Lanreotide is administered as a deep SC injection at a dose of 120 mg every 4 weeks (30). Both SSAs are well tolerated, although they may also lead to increased rates of biliary stones so abdominal imaging by ultrasound is recommended every 6 months.

A carcinoid crisis is very rare and can occur when massive amines are released by NET tumors, leading to hypotension and flushing. This can occur in NET patients as a secondary effect to an operative procedure or general anesthesia (31). Most surgeons or interventional radiologists require patients to be pre-medicated with a SSA prior to a procedure to avoid such complications.

In addition to their established role in symptom control, there are now randomized trials demonstrating that SSAs have an antiproliferative effect. The PROMID trial is a randomized phase III trial in 86 patients with midgut NETs, 40% of which are functional (secretory) tumors (25). Patients were randomized to receive either octreotide LAR 30 mg or placebo (25). Time to progression (TTP), the primary endpoint, was significantly increased with octreotide, at 14.3 months compared to 6 months with placebo (HR = 0.34, *p* = 0.000072). The CLARINET trial is a randomized phase III trial in 204 somatostatin receptor-positive patients with non-functioning (non-secretory) well or moderately differentiated-NETs of the pancreas, midgut or hindgut. Patients were randomized to either lanreotide 120 mg SC or placebo (26). Median progression-free survival (PFS), the primary endpoint, was significantly increased in patients who received the lanreotide, at an estimated 24 months as compared to 18 months for placebo (HR = 0.47, *p* < 0.001). A comparison of the PFS in the placebo arms of PROMID and CLARINET (6 and 18 months, respectively) suggests key differences in patient populations, making cross trial comparison impossible. However, both trials illustrated that SSA treatment in patients with NETs incurs an anti-proliferative effect that improves survival in both non-functional and functional pancreatic and other GI NETs. Neither the PROMID nor CLAIRNET trials included any lung NET patients. Results from the LUNA-randomized trial, which was specifically designed for lung and thymic NETS, were recently presented (28). LUNA-randomized patients to pasireotide, everolimus, or a combination of both agents, and all three arms had a promising progression-free rate at 9 months. LUNA confirms that SSA is a viable treatment option for patients with functional lung NETs as they are effective in controlling symptoms and provide antiproliferative benefits. In some jurisdictions, they are approved only for patients who are symptomatic.

### Peptide Receptor Radioligand Therapy

Peptide receptor radioligand therapy specifically delivers a radiolabeled agent to a target, such as somatostatin receptors which often overexpressed on the surface of metastatic lung NETs (27). PRRT using yttrium Y-90 labeled octreotide was first used to treat this disease in the early 1990 and has been delivered and used in many centers for decades, despite the lack of phase III trials confirming benefit.

This has now changed with the results of the phase III NETTER-1 trial (32). This trial enrolled carcinoid patients whose disease was progressing on a standard dose of octreotide 30 mg LAR. Two hundred and thirty patients with grade 1–2 metastatic midgut NETs were randomized to receive either PRRT ^177^Lu-Dotatate, 7.4 GBq every 8 weeks (×4 administrations), or octreotide LAR 60 mg every 4 weeks. The primary endpoint of PFS was not reached for ^177^Lu-Dotatate and was 8.4 months in the control group (HR 0.21, *p* < 0.0001). The objective radiographic response rate was 18% with ^177^Lu-Dotatate and 3% with control (*p* = 0.0008). Overall survival analysis, although preliminary, was positive as well (13 deaths in ^177^Lu-Dotatate group and 22 in control group; *p* = 0.019). The safety profile of PRRT was favorable. Although this trial was conducted primarily in patients with midgut NET, the results may apply to lung NETs that are receptor-positive by nuclear imaging. A retrospective study which included 89 lung NET’s treated with PRRT revealed a response by RECIST in 28% supporting this treatment as an option for pulmonary NETs (33).

### Systemic Therapy: m-TOR Inhibition

As lung NETs have shown increased activation of the mammalian targets of the rapamycin (m-TOR) signaling pathway (34), everolimus, an m-TOR inhibitor, is another potential therapy for lung NET patients. The phase III RADIANT-2 trial evaluated everolimus plus octreotide-LAR compared to octreotide-LAR alone in advanced NETs with carcinoid syndrome (35). Although the trial included patients with lung NETs, it did not stratify by site. Patients treated with dual agents everolimus and octeotride-LAR, experienced a non-significant improvement in PFS of 16.4 months as compared to 11.3 months with octeotride-LAR alone (*p* = 0.026). The predetermined PFS significance rate was 0.0246, so with a *p* value of 0.026, RADIANT-2 missed its mark. In an exploratory subgroup analysis for lung NETs only (*n* = 44), there was a trend toward improved PFS (13.6 months) for dual treatment as compared to 5.6 months for octeotride alone (*p* = 0.228). As RADIANT-2 included only small numbers of patients and was not stratified per site, the trial had to be repeated to test the effect of everolimus without octeotride in a population of patients with non-functional tumors.

**Figure 2:**
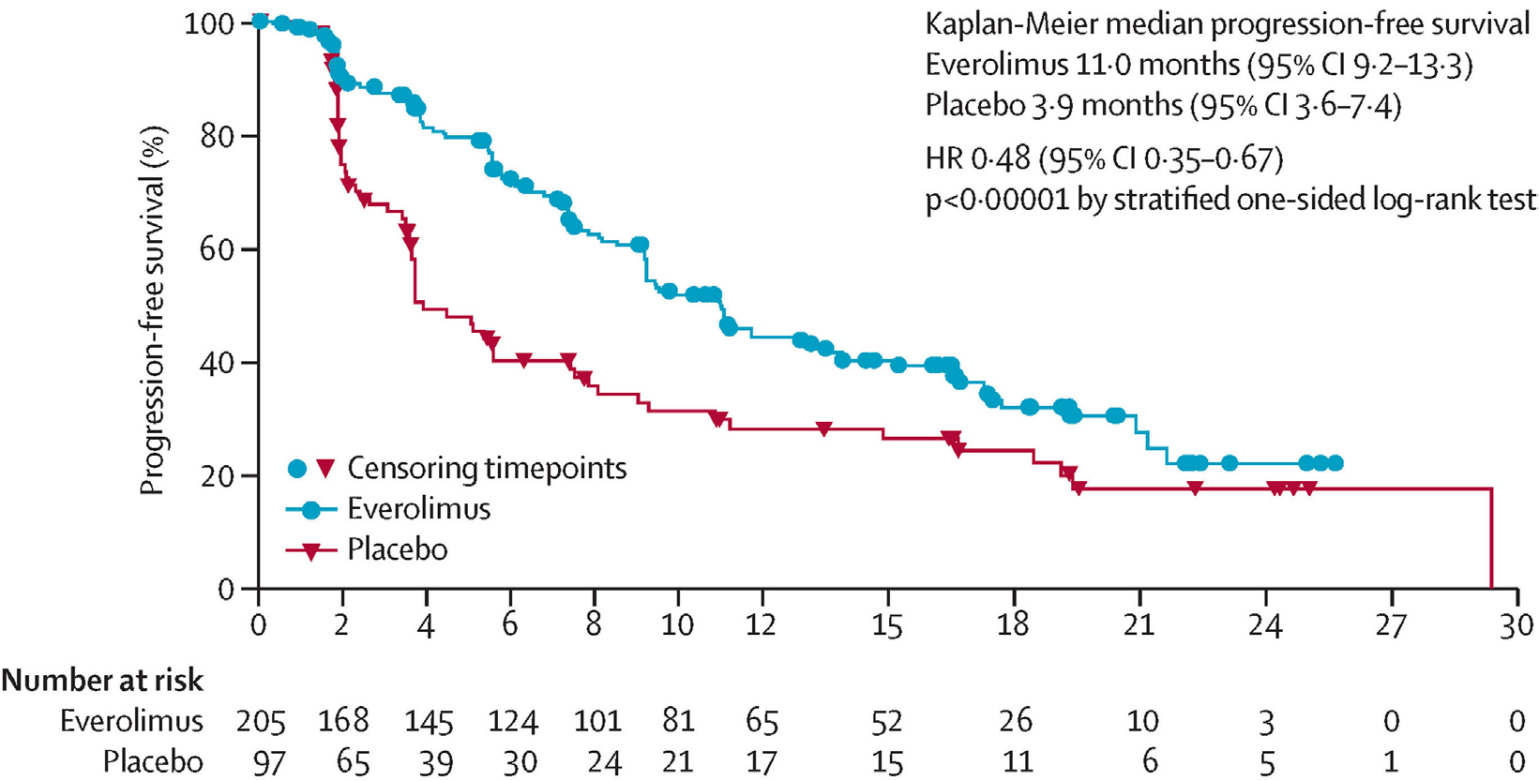
Progression-free survival curve from Radiant 4 trial. PFS curve from the RADIANT 4 trial. Reprinted from Yao et al. (36), Copyright 2016, with permission from Elsevier.

The RADIANT-4 trial randomized patients with non-functional NETs of the lung and GI tract to either everolimus or placebo (36). The median PFS was significantly prolonged in the everolimus arm compared to placebo arm (11 months versus 3.9 months, *p* < 0.00001) (see Figure 2). These improvements were independent of site of disease origin: lung, GI, or unknown.

The phase III RADIANT-2 trial (comparing everolimus and octeotride with octeotride alone) included functional tumors in both lung and GI, and demonstrated that the combination of everolimus and octreotide was not only safe but complementary. However, as the RADIANT-4 trial (comparing everolimus with placebo) excluded functional tumors, the Health Canada label limits everolimus to be used without octeotride for the treatment of non-functional lung NETS only.

### Treatment of NETs Side Effects

Carcinoid heart disease may occur up to 50% of patients with functional tumors (37) and is secondary to serotonin acting on serotonin receptors on the right heart. An echocardiogram can show thickening of the tricuspid valve and surgical management may be needed. The medical management may include diuretics and SSAs to reduce levels of serotonin (38).

### Importance of Multidisciplinary Management

As patients with both TC and AC tumors have prolonged survival and treatment spans across many areas such as surgery, nuclear medicine, medical and radiation oncology, a multidisciplinary approach and or team may be in the patients’ best interest.

## Follow-up

Patients diagnosed with low-grade lung NETs need to be frequently followed up after surgical resection. For patients with TC NETs, conventional imaging can be carried out at 3 and at 6 months, then on an annual basis. For AC, closer monitoring is recommended, first at 3 and 6 months, then continuing at 6-month intervals (3). Clear instructions for the type and interval of follow-up for patients with advanced well-differentiated NETs do not exist (1, 24, 39). Follow-up and imaging needs to be individualized as it is based on the individual baseline status, new symptoms, prior treatment and if change in therapy is contemplated. Chromogranin A measurements can be used to monitor disease progression; however, the frequency and duration of measurement is not articulated. More detailed guidelines are needed to direct follow-up.

## Conclusion

Lung NETs are a unique tumor entity. As the second most common type of NETs, they deserve attention. This heterogenous group of tumors requires a multimodality team approach for optimal treatment. A pathological review is critical to differentiate between low-grade TC and AC NETs and high-grade tumors, and radiologicial imaging is necessary to visualize the tumor and determine metastatic spread. Treatment with somatostatin receptor analogs octreotide and lanreotide can improve carcinoid symptomology and prolong PFS. Tumors that are receptor avid by octreotide may be treated with PRRT with the goal of improving PFS. Finally, m-TOR inhibitors have demonstrated efficacy toward NETs regardless of functional status. The rarity of the disease limits our knowledge, and there is a need for more trials involving lung NET patients. Until more lung-specific data are available, we will have to extrapolate data from the GI NET studies. We look forward to the global understanding of lung NET’s expanding, and the disease finally receiving the attention it deserves.

## Author Contributions

BM reviewed the literature and wrote this article. She is responsible for the content.

## Conflict of Interest Statement

BM has received honoraria from Boehringer Ingelheim, Eli Lilly, Pfizer, Roche, Merck, Bristol-Myers Squibb, Novartis, and AstraZeneca. She is in a consulting/advisory role with Boehringer Ingelheim, her institution has received research funding from Roche and Bayer, and she has received travel/accommodations/expenses from Boehringer Ingelheim, AstraZeneca, Novartis, and Pfizer.

## Abbreviations

AC, atypical carcinoid; DOPA, dihydroxyphenylalanine; 5-HIAA, 5-hydroxyindoleacetic acid; HPF, high power field; HR, hazard ratio; IM, intramuscularly; NETs, neuroendocrine tumors; LAR, long-acting release; NR, non-response; OS, overall survival; PET, positron emission tomography; PFS, progression-free survival; PPRT, peptide receptor radioligand therapy; SC, subcutaneous; SSAs, somatostatin analogs; TC, typical carcinoid; TTP, time to progression; WHO, World Health Organization.
